# Microplastics in the Center of Mediterranean: Comparison of the Two Calabrian Coasts and Distribution from Coastal Areas to the Open Sea

**DOI:** 10.3390/ijerph182010712

**Published:** 2021-10-13

**Authors:** Alessandro Marrone, Mauro F. La Russa, Luciana Randazzo, Daniele La Russa, Emilio Cellini, Daniela Pellegrino

**Affiliations:** 1 Department of Biology, Ecology and Earth Sciences, University of Calabria, 87036 Rende, Italy; mauro.larussa@unical.it (M.F.L.R.); luciana.randazzo@unical.it (L.R.); danielapellegrino@unical.it (D.P.); 2 Department of Pharmacy, Health and Nutritional Sciences, University of Calabria, 87036 Rende, Italy; daniele.larussa@unical.it; 3 Regional Agency for the Environment (ARPACAL), Regional Marine Strategy Centre (CRSM), 88100 Catanzaro, Italy; e.cellini@arpacal.it

**Keywords:** plastic pollution, microplastic polymeric composition, Mediterranean Sea, Calabrian coast, marine strategy

## Abstract

Plastic is everywhere—increasing evidence suggests that plastic pollution is ubiquitous and persistent in ecosystems worldwide. Microplastic pollution in marine environments is particularly insidious, as small fragmentation can increase interaction with biota and food chain access. Of particular concern is the Mediterranean Sea, which has become a large area of accumulation of plastic debris, including microplastics, whose polymeric composition is still largely unknown. In this study, we analyzed the polymeric composition, particle size distribution, shape, and color of small plastic particles (ranging from 50 to 5000 µm) collected from the sea surface in six stations at the center of the Mediterranean Sea. We also described, for the first time, the different distribution of microplastics from coastal areas up to 12 nautical miles offshore. The microplastic density was 0.13 ± 0.19 particles/m^2^, with a marked prevalence of smaller particles (73% < 3 mm) and a peak between 1 and 2 mm (34.74%). Microplastics composition analysis showed that the most abundant material was polyethylene (69%), followed by polypropylene (24%). Moreover, we reported a comparison of the two Calabrian coasts providing the first characterization of a great difference in microplastic concentration between the Tyrrhenian and Ionian sides (87% vs. 13%, respectively), probably due to the complex marine and atmospheric circulation, which make the Tyrrhenian side an area of accumulation of materials originating even from faraway places. We demonstrate, for the first time, a great difference in microplastic concentration between Tyrrhenian and Ionian Calabrian coasts, providing a full characterization and highlighting that microplastic pollution is affected by both local release and hydrography of the areas.

## 1. Introduction

Plastic pollution represents one of the main global environmental concerns of the 21st century due to its transboundary distribution and persistence in ecosystems. It is estimated that more than 5 trillion plastic pieces, weighing over 250,000 tons, have accumulated at sea [1]. All areas of the world are vulnerable to plastic pollution, as the degradation of plastics in seawater is very slow, and during this period, both long-distance transport and continuous degradation occur. Indeed, the environmental conditions in the seas and oceans (water salinity, solar radiation, mechanical degradation) reduce the plastic debris into ever smaller fragments, promoting interactions with the biota [2,3,4,5]. In particular, plastic fragmentation leads to the formation of small plastic particles called microplastics (MPs). The term “microplastics” has been defined differently by various researchers, but currently, MPs are defined as microparticles <5 mm in size, recognizing 333 μm as a practical lower limit when using neuston nets for sampling [6,7]. Since 2011, the most widely used terms from a dimensional point of view are meso-plastics (>5000 µm), micro-plastics (50–5000 µm), and nano-plastics (<50 µm), each with its own set of physical characteristics and biological impacts [8].

Although terrestrial sources, including beach litter, contribute to more than 80% of the plastic debris, the final destination is always the sea. Indeed, MPs are present in all marine environments, even in the most remote areas far from human activities [9,10], from surface to sediment layer [11,12,13,14], as deep as 4844 m in the Porcupine abyssal plain [15] and also in different levels of the food web [16,17,18]. These small plastic fragments, in addition to exerting a direct deleterious effect on marine organisms (alterations in eating behavior, gastrointestinal wounds), act as a vector of various toxic additives and pollutants (both organic and metal pollutants), which can be added during manufacture or adsorbed from the surrounding aquatic environment [19] and thus provide habitats for a wide range of rafting organisms and microbial communities (diatoms, bacteria, and fungi), collectively known as the plastisphere [20]. Due to their small size and high persistence, particulate plastics and associated toxic trace elements are readily ingested and accumulated in many aquatic and terrestrial organisms [21].

Together with the main five oceanic gyres, the Mediterranean Sea has been proposed as the sixth large accumulation zone for marine litter [1,22,23]. This great accumulation of floating plastic is probably related to the hydrodynamics of the Mediterranean Sea, a semi-enclosed basin with outflow mainly occurring through a deep water layer [23]. Indeed, the Mediterranean Sea acts as a convective basin, and it has been suggested that this hydrodynamic pattern involves the retention of local plastic pollution but also the entry of floating plastic pollution from the Atlantic Ocean [24,25]. In addition, in spite of this floating plastic abundance, no equivalent average amount of MPs has been found in the Mediterranean surface waters, suggesting the formation of accumulation areas and/or a particularly high removal rate of small plastic particles by planktivorous animals and/or ballasting by biofouling [8,14,23]. The polymeric composition of the plastic fragments also influences their distribution and segregation in the surface waters, in the water column, in coastal and deep-sea sediments. In fact, depending on their specific density, polymers persist for more or less long from the surface to deep water and therefore have different interactions with the biota [26]. In addition, specific polymers can contain specific additives used during plastic production, and their degradation can significantly alter their ecotoxicological profile [27].

The above evidence points to the need for a MPs’ full characterization through constant monitoring of the Mediterranean to define a polymeric distribution map for plastic pollution.

In this study, six MP collection stations located on the Calabrian coasts were sampled and analyzed, providing the full polymeric characterization of floating MPs and the particle-size distribution, shape, and color of small plastic particles in the center of the Mediterranean Sea. In addition, we described the different distribution of MPs from coastal areas to the open sea, reporting significant features for marine plastic and MPs’ effective management. Lastly, we compared the two Calabrian coasts demonstrating a great difference in MP concentrations between the Tyrrhenian and Ionian sides.

## 2. Materials and Methods

### 2.1. Study Area

**Figure 1:**
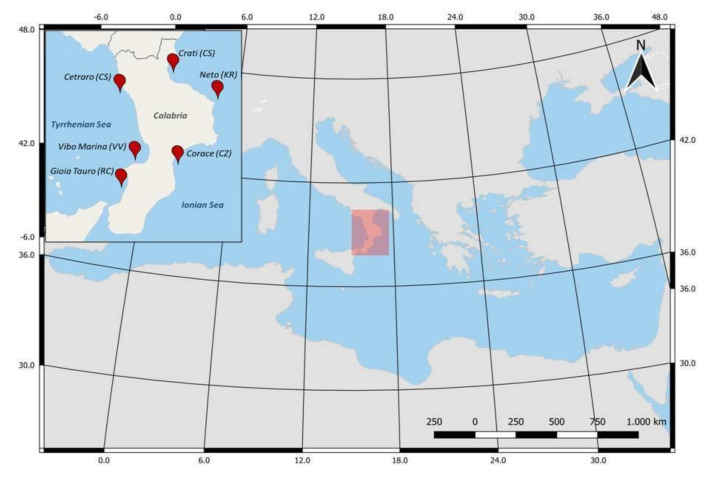
Sampling stations in Ionian (Corace, Neto, and Crati) and Tyrrhenian (Gioia Tauro, Vibo Marina, and Cetraro) coasts of Calabria.

Calabria, with a coastline of about 740 km, is the toe of Italy’s boot and can be considered approximately the center of the Mediterranean basin. We used the sampling areas of the Regional Agency for the Protection of the Environment (ARPA) Calabria Marine Strategy Operational Plan (2019). To have a representative evaluation of the entire region, the sampling sites were chosen in both the Ionian and Tyrrhenian coasts. The choice of areas took into account factors including the distance from sources of direct entry, such as river mouths, port structures, or significant urban settlements, as well as upwelling and downwelling areas or accumulation areas for local hydrodynamic conditions. For each area, samples were taken at 3 stations located at 0.5, 1.5, and 6 nautical miles (NM) from the coast along orthogonal transects. The six sampling stations were as follows (from south to north): Corace (Catanzaro), Neto (Crotone), and Crati (Cosenza), Ionian side; Gioia Tauro (Reggio Calabria), Vibo Marina (Vibo Valentia), and Cetraro (Cosenza), Tyrrhenian side (Figure 1).

### 2.2. Sample Collection and Preparation

The sampling methods were carried out following the guidelines of the Monitoring Programs for the Marine Strategy (Art. 11, Legislative Decree 190/2010) of the European Marine Strategy Directive (2008/56/EC) in order to use the standardized MP monitoring methods. Samples were collected during spring 2020 (May and June). MPs were sampled using a 2.5 m long manta trawl net of 333 μm mesh size with a rectangular frame opening of 25 × 50 cm. The manta towed at the sea surface, in the opposite direction to the current, for 20 min from the ship’s starboard side at an average speed of 2.5 knots. To avoid the wake turbulence, all samplings were made from the ship’s starboard side, beyond the bow wave. After each tow, the net was rinsed with seawater, and subsequently, the collected material was screened by means of two stainless-steel sieves stacked with a mesh vacuum of 5 mm and the underlying one of 300 µm. The accumulated residues were transferred in a glass vial with 70% alcohol for subsequent laboratory analysis.

### 2.3. Microplastics Quantification and Characterization

In the laboratory, the samples were visually inspected under a stereomicroscope (Zeiss Axiolab microscope equipped with a digital camera to capture images), and, using laboratory tweezers, the suspected MP particles were carefully collected and separated from other organic residues. Several previously established criteria (Hidalgo-Ruz et al. (2012); Lusher et al. (2013)) were taken into consideration in order to classify a potential MP particle: (1) absence of cellular or organic structures; (2) a homogenous thickness across the particles; (3) homogenous colors. Once isolated, the potential MPs were counted, photographed and their maximum length (mm), shape, and color were recorded. All samples were examined and double-checked by two different investigators to confirm MP count was consistent and conservative. In order to confirm the polymeric nature of the specimens (suspected MPs) and to allow the specific identification of the different plastic types, all the samples were analyzed by FTIR investigations. Analyses were performed using a PerkinElmer Spectrum 100 spectrophotometer equipped with an attenuated total reflectance (ATR) accessory. Infrared spectra were recorded in ATR mode, in the range of 500–4000 cm^−1^ at a resolution of 4 cm^−1^. Following background scans, 16 scans per particle were performed, and CO_2_ interference was removed for clarity. The obtained spectra were then compared with a library of standard polymeric spectra and accepted with a similarity threshold of more than 70%, in line with suggestions from previous studies (Wakkaf et al., 2020).

### 2.4. Contamination Prevention

Strict quality control measures were taken from beginning to end of the methodological process in order to ensure MP measurements reported are accurate and not artifacts of background contamination in the field or laboratory. We collected a field blank during each sampling event, and then we processed it, in which every step, in the same way as the real samples. During laboratory analysis, as per general control rules, personnel wore 100% cotton laboratory coats and nitrile gloves. In addition, all work surfaces were thoroughly cleaned, and all laboratory equipment (such as sieves, tweezers, and glassware) was rinsed with bi-distilled water. To ensure the non-contamination of our samples with airborne synthetic fibers, a procedural blank consisting of clean Petri dishes (as control) was used in parallel to the samples’ analysis. Examination of blanks samples, in the field, and laboratory, was not found to significantly affect the results of the present work.

## 3. Results and Discussion

### 3.1. Microplastic Abundance, Size, Shape, and Color

Our research confirms that the Mediterranean Sea, similar to most of the seas in the world, is heavily contaminated with MPs. Indeed, MPs collected at all stations show a mean density of 0.13 ± 0.19 particles/m^2^ (0.52 ± 0.77 particles/m^3^) and a density range from 0.01 to 0.66 particles/m^2^. A comparison of our results and other reports both in the Mediterranean and in other seas is shown in Table 1. As different MP sampling methodologies were used, we selected published reports that used sampling methods similar to ours. As evident, there is great variability in the MP concentration even in territorially nearby areas. It was not possible for us to compare the type of plastics sampled in the various areas as most of these studies do not analyze the MPs’ polymeric composition.

A new and significant feature that emerged from our study is the different distribution of MPs from coastal areas to the open sea. Analyzing the density of the MPs detected at different sampling distances from the coast (0.5, 1.5, and 6 NM), we found that almost 50% of the sampled MPs result from areas 0.5 NM distant from the coast and exactly 0.5 NM, 48%; 1.5 NM, 24%; 6NM, 28%. MP abundance and distribution were determined by both anthropogenic and environmental factors. Marine MPs predominantly originate near the coast due to human activities, but environmental factors (wave currents, tides, cyclones, wind directions, river hydrodynamics) may well play a major role in their distribution [28]. In addition, MP transportation pathways are also characterized by complex dynamics due to changes in physiochemical characteristics, processes of biofouling, further fragmentation, aggregation, and biota interactions [29,30,31]. However, the movement of MPs from coastal areas to the deep ocean and vice versa is poorly explored, while understanding the fate and transport of MPs is an important factor to determine the entry point for MPs into the food chain.

**Figure 2:**
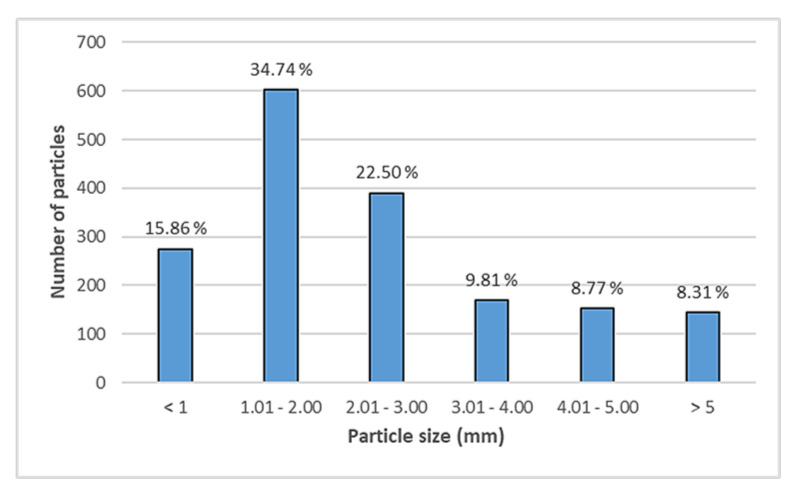
Particle-size distribution of microplastics in the center of the Mediterranean Sea.

Particle size is a key factor in interactions with marine organisms and in particular with their ingestion. The comprehensive size–class distribution of our samples showed a marked prevalence of smaller particles (73% until 3 mm), with a peak between 1 and 2 mm in size (34.74%), while the largest MPs (between 3 and 5 mm) were less than 18% (Figure 2). Despite the use of a filter with pores <5 mm, we also found particles larger than 5 mm, as these MPs were essentially lines or fibers, which, thanks to their elongated shape, passed through the filter pores. This result is not surprising as, particularly in the marine environment, plastic debris degrades into smaller and smaller fragments, increasing in number as their size decreases [32]. This phenomenon has been observed in various marine environments, both in the seas and in the oceans [31,32,33,34]. Only 8% of the total plastic particles were larger than 5mm (Figure 2); thus, our data confirm that over 92% of all plastic items found at sea are commonly smaller than 5 mm [1]. This evidence is particularly alarming, as the size of MPs is a crucial factor for their damaging effects on aquatic life, the smaller the particle size is, the higher are the chance of ingestion and the retention rate by organisms [35,36]. Indeed, uptake, retention, and location of MPs in organisms are closely connected to their size, as the number of particles found in the various organs increases with decreasing particle size [36].

**Figure 3:**
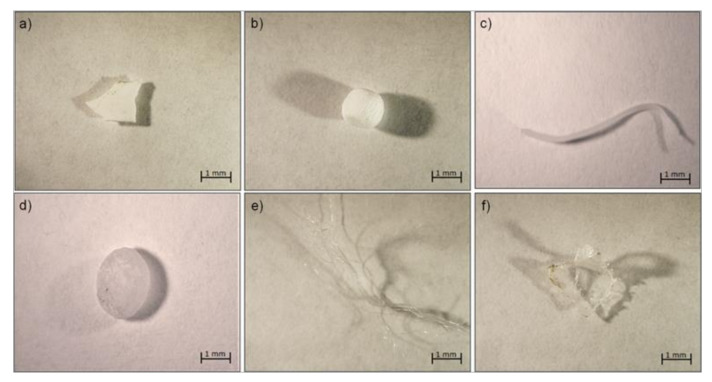
Examples of microplastics with different sizes and shapes: (**a**) fragment, (**b**) foam, (**c**) line, (**d**) pellet, (**e**) fiber, (**f**) film.

**Figure 4:**
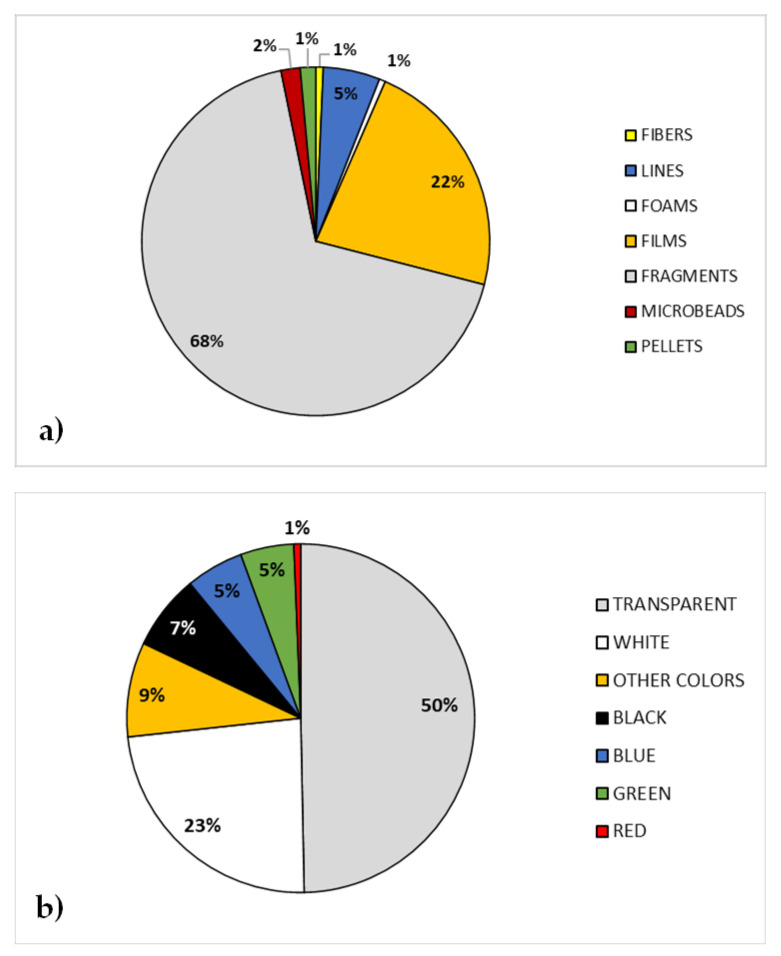
Shape (**a**) and color (**b**) of microplastics in the center of the Mediterranean Sea.

Another determining factor for MPs’ hazardous effect is their shape. In our sampling, according to their appearances and features, the MPs were categorized into seven different shapes: fiber, line, foam, film, fragment, microbeads, and pellet (examples of MPs with different sizes and shapes are shown in Figure 3). Irregular fragments are the most abundant and represent 68% of total plastics, film, and lines accounted for 22% and 5%, respectively, whereas all the other shapes reach 5% all together (Figure 4a). With the exception of pellets and beads, which are primary MPs (native MPs), the other specimens sampled were secondary MPs that originated in the long-term marine environment through photochemical, mechanical, and biological processes [2]. In fact, during field sampling, plastic bottles and bags, fishing nets, and food wrappers were often observed on both beaches and seawater. In particular, hard plastic and outer packaging might be the source of fragments, plastic bags might be the main source of films [13,14], while lines mainly derived from broken fishing lines. Once in the seawater, plastic lines sink to the sea bottom, and only a small part remains suspended in the water column [37], which explains why few lines were collected. Although fibers represent only 1% of our samples, this type of MPs should be given the highest attention, both for their toxicity and for their sampling difficulty. Indeed, several studies suggest that the fibers have a particularly insidious shape for aquatic organisms, with interference both in the gut and in the gills, resulting in acute toxicity and high mortality [35,36]. Hence, their toxicity may be caused by the MPs themselves, the additives that they contain, and/or by other chemical compounds adsorbed by seawater [38,39]. In addition, the amount of fiber could be much higher than reported, as the sampling methods at sea currently used (towed nets generally 300–350-μm mesh) may underestimate the amount of microfibers present, which may pass through the mesh due to their narrow width [40].

MPs occur in a range of colors [41]. MPs’ color is not a secondary factor for their threat to marine life, as the color affects the interaction with marine organisms—some colors seem to affect the ability to distinguish between plastics and their natural food [42,43]. Aquatic organisms ingest MPs together with their food, deceived by their size and especially by their color; indeed, small plastic fragments have been reported in several species and in all phases of the marine food chain [44,45,46]. According to literature data, transparent and white were the most common particles in our sampling and accounted for 50% and 23%, respectively, whereas colorful plastics (black, blue, green, red, and other colors) accounted for the remaining 27% (Figure 4b). Therefore, our results showed a very high percentage of MPs with color characteristics (73%) that promote their entry into the food chain of aquatic organisms. An important source of these types of particles (transparent and white) might be plastic bags, extensively used in daily life. Furthermore, this prevalence of colorless particles could also be due to the loss of color that many colored particles, especially some lines and films, undergo once they enter the surface waters. In turn, some white plastics had turned pale yellow and, not surprisingly, showed rounded corners, due to environmental exposure over a long period of time. 

### 3.2. Microplastic Composition

**Figure 5:**
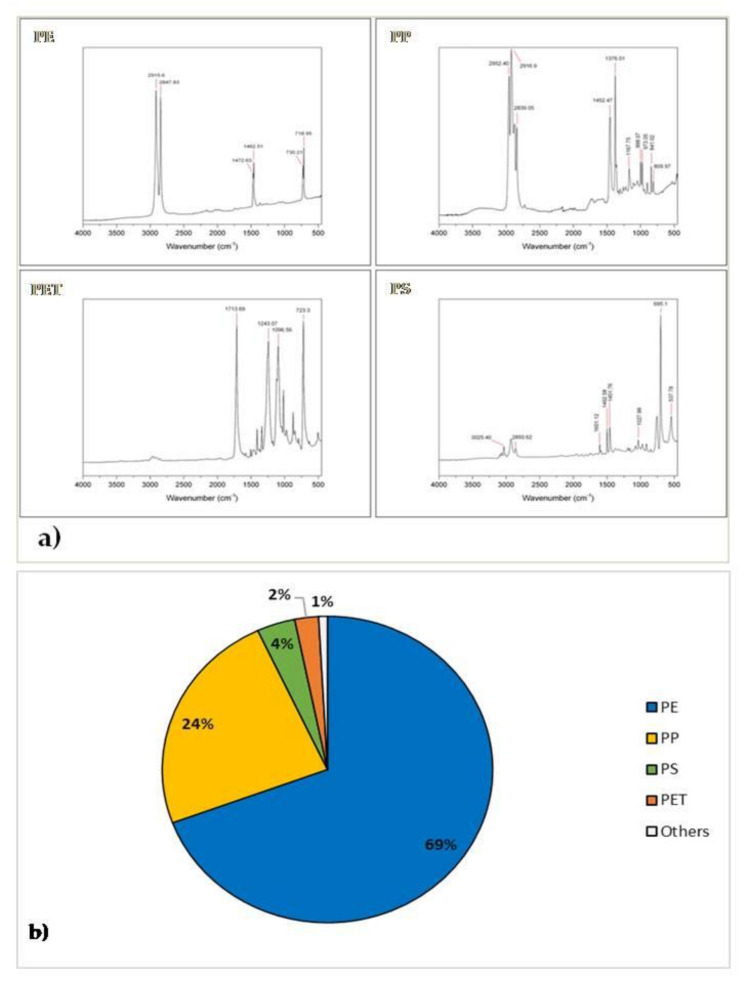
(**a**) Identification of microplastics using a Fourier transform infrared spectroscope; (**b**) polymeric composition of all particles characterized through ATR FTIR analysis (PE, polyethylene; PP, polypropylene; PET, polyethylene terephthalate; PS, polystyrene).

The polymeric identity of all plastic particles (n = 1733) was verified through ATR FTIR (Figure 5a). Composition analysis of MPs showed that the most abundant material was polyethylene (PE), which accounted for 69%, followed by polypropylene (PP) (24%), whereas polystyrene (PS), polyethylene terephthalate (PET), and other types of polymers accounted for the remaining 7% (Figure 5b). The polyolefins, which include PE and PP, are a family of thermoplastics produced mainly from oil and natural gas through a polymerization process of ethylene and propylene, respectively. Their versatility has made them the most produced polymers worldwide [47]. PE and PP, with excellent mechanical properties, heat resistance, stable chemical properties, and good electrical insulation, are widely used for food and medical packaging, bottles (for drinks, detergents, cosmetics), toys, pipes, and household utensils. A disadvantage of PP, compared with PE, is its poor resistance to UV rays and oxidation [30,48]; therefore, PP ages faster in the ocean environment and easily breaks down into smaller particles. Most of the studies carried out, including ours, report a higher concentration of PE MPs, compared with PP, but these data could be distorted by the extreme sensitivity of PP to marine environmental conditions that could lead to the rapid formation of fragments with nanomolar dimensions. Another important factor that may explain the prevalence of these two polymers is their low density, compared with other plastic polymers. The densities of PE and PP (0.95 g/m^3^ and 0.92 g/m^3^, respectively) are lower than seawater, whereby these polymers easily float on the water surface. Being widely used in the disposable packaging industry and having lower densities than seawater, it is not surprising that these polymers consistently account for the majority of the plastic particles floating in the Mediterranean Sea and surface waters worldwide [49,50]. On the contrary, PET and PS have densities greater than that of water, but their presence in marine surface waters has also been reported in several studies [51,52]. This finding could be explained by the fact that MPs’ vertical distribution in seawater depends on density but also on other factors such as hydrodynamic conditions, salinity, temperature, and wind [53]. An important feature that emerged from this study was the absence of a clear correlation/pattern of polymers with size, shape, and color, with the exception of the PET particles, which were all fibers or filaments. Due to its transparency, PET is the most widely used polymer in the bottling sector, but it can also be spun into polyester lines, which is the common raw material for clothes and fishing nets [54].

### 3.3. Microplastic Distribution

**Figure 6:**
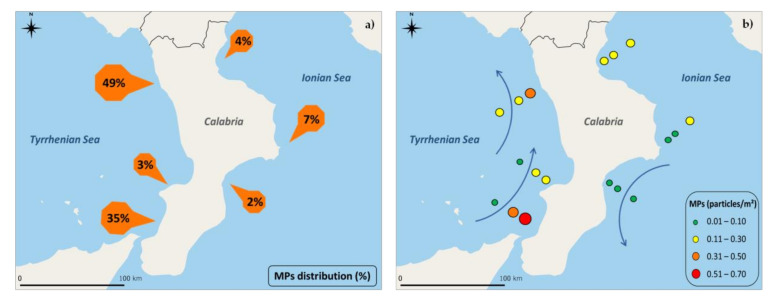
(**a**) Map of the Calabrian coasts showing the location of sampling stations and the distribution of plastic densities expressed as percentages; (**b**) microplastics’ density at different sampling distances from the coast (0.5, 1.5, 6 nautical miles) in the 6 Calabrian sampling sites; blue arrows represent ocean currents.

Analyzing the distribution of the collected MPs at the six sampling sites (three on the Ionian side and three on the Tyrrhenian side of Calabria), it was clear that the highest concentration density (almost 87%) is found on the Tyrrhenian side (Figure 6a). These data are in line with the results of the monitoring of beached waste conducted over a three-year period (2015–2017) by the Regional Environmental Protection Agency of Calabria (Arpacal). Indeed, this study shows firstly that the Calabrian sea coast most subject to the presence of beached waste is the Tyrrhenian side and then highlights that most of this waste, respectively, 90% on the Tyrrhenian and 76% on the Ionian, are plastic material [55]. No wonder the possibility that at least part of this waste, sooner or later, as a result of natural phenomena such as storm surges and high tides, can end up directly into the sea. Clearly, the data on beached waste cannot explain this great difference in the concentration of floating MPs between the two coasts (87% vs. 13%); therefore, additional factors must be involved. On the Tyrrhenian coast, there are major Calabrian commercial ports; indeed, the two Tyrrhenian sampling stations that present almost all of the collected MPs are the two ports, Cetraro and Gioia Tauro, with 49% and 35%, respectively. Cetraro is located in a densely populated holiday area and is an essentially tourist/fishing port, while Gioia Tauro is the largest and most important commercial port in Calabria (and in the Mediterranean for transshipment). It is, therefore, not surprising that these two areas have this high rate of plastic pollution given the high frequency of ships and many tourist activities along their coast. Indeed, anthropogenic activity was found to be a key factor for MP pollution [49,56]. In addition, the abundance of MPs within the ports could also be related to the geometry of the harbor compartments (partially enclosed areas) and their proximity to industrial activities. What is surprising is the other Tyrrhenian site, Vibo Marina, which, despite its proximity to the third commercial port of Calabria, has a concentration of MPs comparable to the Ionian side (3%). This outcome highlights that the presence of ports is not the determining factor of the high degree of pollution of the Tyrrhenian coast, confirming that the spatial distribution of MP concentrations is irregular without a clear association with sources, and this phenomenon could be due to variability, even in the short scale, in the Mediterranean surface circulation [24,57]. The Mediterranean Sea is a semi-enclosed basin connected with the Atlantic Ocean through the Gibraltar Strait, and despite the almost complete closure, it is characterized by a complex marine circulation resulting from intricate interactions and feedback between ocean–atmosphere–land processes that play a prominent role in the climate and hydrological cycle [58]. It has long been known that this Mediterranean hydrodynamic pattern involves the entry of floating plastic pollution from the Atlantic Ocean [24,25], and this less saline ocean current, once it enters the basin, is diverted to the right by the Coriolis force and tends to skirt all coasts in an anticlockwise direction. Furthermore, the prevailing superficial winds blow from the sea to the Tyrrhenian coast, contributing to the accumulation of floating material toward the coastal area. Indeed, the largest accumulation area is represented specifically by the Calabrian Tyrrhenian coast and the Strait of Messina, which orographically represent a funnel both for floating and seabed debris [59,60]. The reverse occurs on the Ionian coast, where both winds and currents tend to move the floating material away toward the open sea. Consequently, it is reasonable to assume that the great difference observed between the two Calabrian coasts can be attributed toa high degree to the complex marine and atmospheric circulation that makes the Tyrrhenian side an area of accumulation of materials originating even from faraway locations.

An additional result supporting this hypothesis is that in the Tyrrhenian sites, the great abundance of MPs shows a decreasing gradient as the distance from the coast increases, while in the Ionian sites, the gradient is the opposite (Figure 6b). This remarkable result can be explained by the different movement of the ocean currents, which are typically felt at 3–4 NM from the coast, therefore between our 1.5 and 6 NM stations, on the two sides of the region (Figure 6b, blue arrows). Several studies confirm the widespread distribution of MPs, reporting their discovery also in remote areas, apparently free from possible input sources, and underline the heterogeneity of dispersion patterns in the open sea, influenced by several factors, including hydrodynamic features such as surface currents, up- and downwelling, eddies, and wave movements [61,62,63]. 

## 4. Conclusions

This study reported MP distribution and characterization in the center of the Mediterranean Sea, comparing the two Calabrian coasts. Although a small-scale study, our results corroborate data reported by other studies previously conducted on a larger scale in the Mediterranean basin. First, our results confirm that the sampled sites are contaminated by various typologies of MPs, with a clear prevalence of PE, compared with other polymers. We also characterized the different distribution of MPs from coastal areas to the open sea, reporting significant features for marine plastic and MPs’ effective management. Moreover, we provided the first characterization of a great difference in MP concentrations between the two Calabrian coasts (Tyrrhenian and Ionic sides), identifying the Tyrrhenian side as a local hotspot, and demonstrating small-scale heterogeneity in plastics distribution, which probably reflects a complex interaction between pollution sources, sinks, and polymers persistence in the aquatic environment. These lines of evidence underline the need for MPs’ full characterization through constant monitoring of the Mediterranean to define full distribution maps for plastic pollution. Long-term extensive monitoring programs should be realized on a regular basis to extract local and overall data, better quantify the MP pollution in spatial and vertical profiles in a broader range of Mediterranean sub-regions, and translate this knowledge into predictive models. Clearly, harmonization of MP monitoring methods and homologation of the sampling protocols to the guidelines of the monitoring programs of the European Marine Strategy Directive (i.e., those used in this study) is, therefore, strongly recommended, in order to allow a reliable and comparable quantitative analysis of the data obtained from different studies. Only the overcoming of these biases will allow obtaining the necessary information to implement knowledge of plastic contamination patterns in the marine environment to realize specific and effective solutions. 

In conclusion, our results provide significant information to plan a monitoring system over time and space, in order to identify the best marine strategy to mitigate the sources of MP pollution.

## Figures and Tables

**Table 1:** Microplastic concentrations in marine waters.

Study Area	Net Mesh	Mean Density	Reference
Mediterranean	200 µm	0.243 particles/m^2^	(Cózar et al., 2015)
North Western Mediterranean	333 µm	0.116 particles/m^2^	(Collignon et al., 2012)
Western Mediterranean	333 µm	0.135 particles/m^2^	(Faure et al., 2016)
Western Mediterranean-Adriatic	200 µm	0.40 (±0.74) particles/m^2^	(Suaria et al., 2016)
Mediterranean-Corsica	200 µm	0.062 particles/m^2^	(Collignon et al., 2014)
Central-Western Mediterranean	500 µm	0.15 particles/m^3^	(de Lucia et al., 2014)
Central-Western Mediterranean	333 µm	0.147 particles/m^2^	(Ruiz-Orejón et al., 2016)
Sardinian Sea	200 µm	0.16 (±0.31) particles/m^3^	(Fossi et al., 2016)
Sardinian Sea	200 µm	0.17 (±0.32) particles/m^3^	(Panti et al., 2016)
Ligurian Sea	200 µm	0.49 (±1.66) particles/m^3^	(Fossi et al., 2016)
Ligurian Sea	333 µm	0.103 particles/m^3^	(Pedrotti et al., 2014)
Calabrian costs	333 µm	0.13 (±0.194) particles/m^2^	This study
		0.52 (±0.778) particles/m^3^	
North Atlantic	350 µm	1.70 particles/m^3^	(Eriksen et al., 2014)
North-east Atlantic	250 µm	2.46 particles/m^3^	(Lusher et al., 2014)
East Asian seas	335 µm	3.70 (±10.4) particles/m^3^	(Isobe et al., 2015)
Seto Inland Sea	335 µm	0.39 particles/m^3^	(Isobe et al., 2014)
Arctic polar waters	333 µm	0.34 (±0.31) particles/m^3^	(Lusher et al., 2015)
Bohai Sea	330 µm	0.33 (±0.36) particles/m^3^	(Weiwei et al., 2017)

## References

[1] Eriksen M., Lebreton L.C.M., Carson H.S., Thiel M., Moore C.J., Borerro J.C., Galgani F., Ryan P.G., Reisser J. (2014). Plastic Pollution in the World’s Oceans: More than 5 Trillion Plastic Pieces Weighing over 250,000 Tons Afloat at Sea. PLoS ONE.

[2] Wang G.X., Huang D., Ji J.H., Völker C., Wurm F.R. (2021). Seawater-Degradable Polymers—Fighting the Marine Plastic Pollution. Adv. Sci..

[3] Zhang K., Hamidian A.H., Tubić A., Zhang Y., Fang J.K.H., Wu C., Lam P.K.S. (2021). Understanding plastic degradation and microplastic formation in the environment: A review. Environ. Pollut..

[4] Cai L., Wu D., Xia J., Shi H., Kim H. (2019). Influence of physicochemical surface properties on the adhesion of bacteria onto four types of plastics. Sci. Total Environ..

[5] Fotopoulou K.N., Karapanagioti H.K. (2015). Surface properties of beached plastics. Environ. Sci. Pollut. Res..

[6] Fendall L.S., Sewell M.A. (2009). Contributing to marine pollution by washing your face: Microplastics in facial cleansers. Mar. Pollut. Bull..

[7] Arthur C., Baker J., Bamford H. (2009). Proceedings of the International Research Workshop on the Occurrence, Effects, and Fate of Microplastic Marine Debris, Tacoma, WA, USA, 9–11 September 2008.

[8] Andrady A.L. (2011). Microplastics in the marine environment. Mar. Pollut. Bull..

[9] Obbard R.W., Sadri S., Wong Y.Q., Khitun A.A., Baker I., Thompson R.C. (2014). Global warming releases microplastic legacy frozen in Arctic Sea ice. Earth’s Futur..

[10] Reed S., Clark M., Thompson R., Hughes K.A. (2018). Microplastics in marine sediments near Rothera Research Station, Antarctica. Mar. Pollut. Bull..

[11] Zhang D., Liu X., Huang W., Li J., Wang C., Zhang D., Zhang C. (2020). Microplastic pollution in deep-sea sediments and organisms of the Western Pacific Ocean. Environ. Pollut..

[12] Graham E.R., Thompson J.T. (2009). Deposit- and suspension-feeding sea cucumbers (Echinodermata) ingest plastic fragments. J. Exp. Mar. Bio. Ecol..

[13] Nor N.H.M., Obbard J.P. (2014). Microplastics in Singapore’s Coastal Mangrove Ecosystems. Mar. Pollut. Bull..

[14] Thompson C.R., Olsen Y., Mitchell P.R., Davis A., Rowland J.S., John W.G.A., McGonigle D., Russell E.A. (2004). Lost at Sea: Where Is All the Plastic?. Science.

[15] Van Cauwenberghe L., Vanreusel A., Mees J., Janssen C.R. (2013). Microplastic pollution in deep-sea sediments. Environ. Pollut..

[16] Dantas D.V., Barletta M., da Costa M.F. (2012). The seasonal and spatial patterns of ingestion of polyfilament nylon fragments by estuarine drums (Sciaenidae). Environ. Sci. Pollut. Res..

[17] Eriksson C., Burton H. (2003). Origins and Biological Accumulation of Small Plastic Particles in Fur Seals from Macquarie Island. Ambio.

[18] Jantz L.A., Morishige C.L., Bruland G.L., Lepczyk C.A. (2013). Ingestion of plastic marine debris by longnose lancetfish (*Alepisaurus ferox*) in the North Pacific Ocean. Mar. Pollut. Bull..

[19] Selvam S., Jesuraja K., Venkatramanan S., Roy P.D., Jeyanthi Kumari V. (2021). Hazardous microplastic characteristics and its role as a vector of heavy metal in groundwater and surface water of coastal south India. J. Hazard. Mater..

[20] Sharma S., Sharma V., Chatterjee S. (2021). Microplastics in the Mediterranean Sea: Sources, Pollution Intensity, Sea Health, and Regulatory Policies. Front. Mar. Sci..

[21] Bradney L., Wijesekara H., Palansooriya K.N., Obadamudalige N., Bolan N.S., Ok Y.S., Rinklebe J., Kim K.H., Kirkham M.B. (2019). Particulate plastics as a vector for toxic trace-element uptake by aquatic and terrestrial organisms and human health risk. Environ. Int..

[22] Lebreton L.C.M., Greer S.D., Borrero J.C. (2012). Numerical modelling of floating debris in the world’s oceans. Mar. Pollut. Bull..

[23] Cózar A., Sanz-Martín M., Martí E., González-Gordillo J.I., Ubeda B., Ágálvez J., Irigoien X., Duarte C.M. (2015). Plastic accumulation in the mediterranean sea. PLoS ONE.

[24] Béranger K., Drillet Y., Houssais M.N., Testor P., Bourdallé-Badie R., Alhammoud B., Bozec A., Mortier L., Bouruet-Aubertot P., Crépon M. (2010). Impact of the spatial distribution of the atmospheric forcing on water mass formation in the Mediterranean Sea. J. Geophys. Res. Ocean..

[25] Soto-Navarro J., Criado-Aldeanueva F., García-Lafuente J., Sánchez-Romn A. (2010). Estimation of the Atlantic inflow through the Strait of Gibraltar from climatological and in situ data. J. Geophys. Res. Ocean..

[26] Erni-Cassola G., Zadjelovic V., Gibson M.I., Christie-Oleza J.A. (2019). Distribution of plastic polymer types in the marine environment; A meta-analysis. J. Hazard. Mater..

[27] Schrank I., Trotter B., Dummert J., Scholz-Böttcher B.M., Löder M.G.J., Laforsch C. (2019). Effects of microplastic particles and leaching additive on the life history and morphology of Daphnia magna. Environ. Pollut..

[28] Shahul Hamid F., Bhatti M.S., Anuar N., Anuar N., Mohan P., Periathamby A. (2018). Worldwide distribution and abundance of microplastic: How dire is the situation?. Waste Manag. Res..

[29] Law K.L., Thompson R.C. (2017). Oceans. Microplastics in the seas. Science.

[30] Andrady A.L. (2017). The plastic in microplastics: A review. Mar. Pollut. Bull..

[31] Weiwei Z., Shoufeng Z., Juying W., Yan W., Jingli M., Ping W., Xinzhen L., Deyi M. (2017). Microplastic pollution in the surface waters of the Bohai Sea, China. Environ. Pollut..

[32] Isobe A. (2016). Percentage of microbeads in pelagic microplastics within Japanese coastal waters. Mar. Pollut. Bull..

[33] Song Y.K., Hong S.H., Jang M., Kang J.H., Kwon O.Y., Han G.M., Shim W.J. (2014). Large accumulation of micro-sized synthetic polymer particles in the sea surface microlayer. Environ. Sci. Technol..

[34] Isobe A., Uchiyama-Matsumoto K., Uchida K., Tokai T. (2017). Microplastics in the Southern Ocean. Mar. Pollut. Bull..

[35] Gray A.D., Weinstein J.E. (2017). Size- and shape-dependent effects of microplastic particles on adult daggerblade grass shrimp (*Palaemonetes pugio*). Environ. Toxicol. Chem..

[36] Kögel T., Bjorøy Ø., Toto B., Bienfait A.M., Sanden M. (2020). Micro- and nanoplastic toxicity on aquatic life: Determining factors. Sci. Total Environ..

[37] Engler R.E. (2012). The complex interaction between marine debris and toxic chemicals in the ocean. Environ. Sci. Technol..

[38] (2015). GESAMP Joint Group of Experts on the Scientific Aspects of Marine Environmental Protection Sources, fate and effects of microplastics in the marine environment: A global assessment. Rep. Stud. GESAMP.

[39] Bowmer T., Kershaw P. (2013). Proceedings of GESAMP International Workshop on Microplastic particles as a vector in transporting persistent, bioaccumulating and toxic substances in the ocean. GESAMP Reports Stud..

[40] Covernton G.A., Pearce C.M., Gurney-Smith H.J., Chastain S.G., Ross P.S., Dower J.F., Dudas S.E. (2019). Size and shape matter: A preliminary analysis of microplastic sampling technique in seawater studies with implications for ecological risk assessment. Sci. Total Environ..

[41] Martí E., Martin C., Galli M., Echevarría F., Duarte C.M., Cózar A. (2020). The Colors of the Ocean Plastics. Environ. Sci. Technol..

[42] Boerger C.M., Lattin G.L., Moore S.L., Moore C.J. (2010). Plastic ingestion by planktivorous fishes in the North Pacific Central Gyre. Mar. Pollut. Bull..

[43] Wright S.L., Thompson R.C., Galloway T.S. (2013). The physical impacts of microplastics on marine organisms: A review. Environ. Pollut..

[44] Setälä O., Lehtiniemi M., Coppock R., Cole M. (2018). Microplastics in marine food webs. Microplastic contamination in aquatic environments.

[45] Desforges J.P.W., Galbraith M., Ross P.S. (2015). Ingestion of Microplastics by Zooplankton in the Northeast Pacific Ocean. Arch. Environ. Contam. Toxicol..

[46] Walkinshaw C., Lindeque P.K., Thompson R., Tolhurst T., Cole M. (2020). Microplastics and seafood: Lower trophic organisms at highest risk of contamination. Ecotoxicol. Environ. Saf..

[47] (2019). Plastics Europe Plastics-the Facts 2019. An Analysis of European Plastics Production, Demand and Waste Data. https://www.plasticseurope.org/application/files/9715/7129/9584/FINAL_web_version_Plastics_the_facts2019_14102019.pdf.

[48] Tripathi D. (2002). Practical Guide to Polypropylene.

[49] Hidalgo-Ruz V., Gutow L., Thompson R.C., Thiel M. (2012). Microplastics in the marine environment: A review of the methods used for identification and quantification. Environ. Sci. Technol..

[50] Frias J.P.G.L., Otero V., Sobral P. (2014). Evidence of microplastics in samples of zooplankton from Portuguese coastal waters. Mar. Environ. Res..

[51] Suaria G., Avio C.G., Mineo A., Lattin G.L., Magaldi M.G., Belmonte G., Moore C.J., Regoli F., Aliani S. (2016). The Mediterranean Plastic Soup: Synthetic polymers in Mediterranean surface waters. Sci. Rep..

[52] Enders K., Lenz R., Stedmon C.A., Nielsen T.G. (2015). Abundance, size and polymer composition of marine microplastics ≥10 μm in the Atlantic Ocean and their modelled vertical distribution. Mar. Pollut. Bull..

[53] Zhao S., Zhu L., Li D. (2015). Microplastic in three urban estuaries, China. Environ. Pollut..

[54] Tan X., Yu X., Cai L., Wang J., Peng J. (2019). Microplastics and associated PAHs in surface water from the Feilaixia Reservoir in the Beijiang River, China. Chemosphere.

[55] Cellini E.P.L. (2020). I dati della regione mar ionio mediterraneo centrale. Ecoscienza.

[56] Mani T., Hauk A., Walter U., Burkhardt-Holm P. (2015). Microplastics profile along the Rhine River. Sci. Rep..

[57] Poulain P.M., Bussani A., Gerin R., Jungwirth R., Mauri E., Menna M., Notarstefano G. (2013). Mediterranean surface currents measured with drifters from basin to subinertial scales. Oceanography.

[58] Ducrocq V., Drobinski P., Gualdi S., Raimbault P. (2018). Sub-chapter 1.2.1. The water cycle in the Mediterranean. Mediterr. Reg. under Clim. Chang..

[59] Pierdomenico M., Casalbore D., Chiocci F.L. (2019). Massive benthic litter funnelled to deep sea by flash-flood generated hyperpycnal flows. Sci. Rep..

[60] Canals M., Pham C.K., Bergmann M., Gutow L., Hanke G., van Sebille E., Angiolillo M., Buhl-Mortensen L., Cau A., Ioakeimidis C. (2020). The quest for seafloor macrolitter: A critical review of background knowledge, current methods and future prospects. Environ. Res. Lett..

[61] van der Hal N., Ariel A., Angel D.L. (2017). Exceptionally high abundances of microplastics in the oligotrophic Israeli Mediterranean coastal waters. Mar. Pollut. Bull..

[62] Pedrotti M.L., Petit S., Elineau A., Bruzaud S., Crebassa J.C., Dumontet B., Martí E., Gorsky G., Cózar A. (2016). Changes in the floating plastic pollution of the mediterranean sea in relation to the distance to land. PLoS ONE.

[63] Kazour M., Jemaa S., Issa C., Khalaf G., Amara R. (2019). Microplastics pollution along the Lebanese coast (Eastern Mediterranean Basin): Occurrence in surface water, sediments and biota samples. Sci. Total Environ..

